# Shaping the Future of Respiratory Care: A Look Into the Next Decade and Strategic Recommendations by the European Forum for Research and Education in Allergy and Airways Diseases

**DOI:** 10.1016/j.chest.2025.12.043

**Published:** 2026-01-14

**Authors:** Xander Bertels, Glenis K. Scadding, Vibeke Backer, Susanne Lau, Wytske J. Fokkens, Peter J. Barnes, Manuel Bernal Sprekelsen, Leif Bjermer, Michael Blaiss, Elena Borzova, Marie-Charlotte Brüggen, Guy G. Brusselle, Lars Olaf Cardell, Diego M. Conti, Marjolein Cornet, Eugenio De Corso, Bernard De Groeve, Ratko Djukanovic, Adam T. Fox, Mina Gaga, Philippe Gevaert, Peter Gibson, Claudia L. Gray, Joseph Han, Liam Heaney, Enrico Heffler, Hans Jürgen Hoffmann, Claire Hopkins, Daniel Jackson, Outi Jauhola, Milos Jesenak, Pål Johansen, Ekaterina Khaleva, Basile Landis, Stella Lee, Valerie Lund, Mika Mäkelä, Marinda McDonald, Erik Melén, Joaquim Mullol, Antonio Nieto-García, Ian Pavord, Anju Peters, David Price, Santiago Quirce, Dermot Ryan, Pernilla Sahlstrand-Johnson, Sophie Scheire, Peter Schmid-Grendelmeier, Sven Schneider, Brent A. Senior, Catherine M.E. Shire, Peter K. Smith, Zsolt Szépfalusi, M. Thijs A. Teeling, Michael E. Wechsler, Philippe Houssiau, Klaus F. Rabe, Peter W. Hellings, José Luis Castro

**Affiliations:** a European Forum for Research and Education in Allergy and Airway diseases, Brussels, Belgium; b Upper Airways Research Laboratory, Department of Head and Skin, Ghent University, Ghent, Belgium; c ENT Department, Royal National ENT Hospital, London, England; d Division of Immunity and Infection, University College London, London, England; e Ear Institute, University College London, London, England; f Department of Otorhinolaryngology, Head & Neck Surgery, and Audiology, Rigshospitalet, Copenhagen University, Copenhagen, Denmark; g Department of Pediatric Respiratory Medicine, Immunology and Critical Care Medicine, Charité Universitätsmedizin Berlin, Berlin, Germany; h Amsterdam UMC, Department of Otorhinolarynogology, University of Amsterdam, Amsterdam, the Netherlands; i National Heart and Lung Institute, Imperial College London, London, England; j Hospital Clinic, Barcelona, University of Barcelona, Spain; k Department of Respiratory Medicine & Allergology, Institute for Clinical Science, Skane University Hospital, Lund University, Lund, Sweden; l Medical College of Georgia at Augusta University, Augusta, GA; m Dermatology Division, Niigata University Graduate School of Medical and Dental Sciences, Niigata, Japan; n Christine Kühne-Center for Allergy Research and Education (CK-CARE), Davos, Switzerland; o Department of Dermatology, University Hospital Zurich, Zurich, Switzerland; p Department of Respiratory Medicine, Ghent University Hospital, Ghent, Belgium; q Department of Epidemiology, Erasmus Medical Center Rotterdam, Rotterdam, the Netherlands; r Department of Respiratory Medicine, Erasmus Medical Center Rotterdam, Rotterdam, the Netherlands; s Division of ENT Diseases, Department of Clinical Sciences, Intervention and Technology, Karolinska Institutet, Stockholm, Sweden; t Department of ENT Diseases, Karolinska University Hospital, Stockholm, Sweden; u Escuela de Doctorado UAM, Centro de Estudios de Posgrado, Universidad Autónoma de Madrid, Madrid, Spain; v Allergy and Clinical Immunology Research Unit, Department of Microbiology and Immunology, KU Leuven, Leuven, Belgium; w Department of Microbiology and Immunology, Allergy and Clinical Immunology Research Unit, KU Leuven, Leuven, Belgium; x Department of Otorhinolaryngology, Alrijne Hospital, Leiden, the Netherlands; y UOC Otorinolaringoiatria, Fondazione Policlinico Universitario A. Gemelli IRCCS, Roma, Italy; z imec, Leuven, Belgium; aa School of Clinical and Experimental Sciences, Medical Faculty, University of Southampton and NIHR Southampton Biomedical Research Centre, Southampton; bb Children’s Allergy Service, Evelina London Children’s Hospital, London, England; cc Department of Paediatric Allergy, King’s College London, London, England; dd 1st Respiratory Medicine Department, Hygeia Hospital, Marousi, Greece; ee Department of Respiratory and Sleep Medicine, John Hunter Hospital, Newcastle, NSW, Australia; ff Department of Paediatrics, University of Cape Town, Cape Town, South Africa; gg Department of Otolaryngology & Head and Neck Surgery, Eastern Virginia Medical School, Norfolk, VA; hh Wellcome-Wolfson Institute for Experimental Medicine, Queens University Belfast, Belfast, Ireland; ii Personalized Medicine Asthma and Allergy Unit-IRCCS Humanitas Research Hospital, Rozzano (Milan); jj Department of Biomedical Sciences–Humanitas University, Pieve Emanuele (Milan), Italy; kk Department of Clinical Medicine, University of Aarhus, Aarhus, Denmark; ll Department of Otolaryngology, King’s College London, London, England; mm Department of Pediatrics, University of Wisconsin School of Medicine and Public Health, Madison, WI; nn Institute of Clinical Immunology and Medical Genetics, Department of Pulmonology and Phthisiology, Department of Pediatrics and Adolescent Medicine, Martin, Slovakia; oo Department of Clinical Immunology and Allergology, Jessenius Faculty of Medicine in Martin, Comenius University in Bratislava, University Hospital in Martin, Martin, Slovakia; pp Department of Dermatology, University of Zurich, Zurich, Switzerland; qq Department of Dermatology, University Hospital of Zurich, Zurich, Switzerland; rr Allergy Unit, Department of Dermatology, University Hospital of Zurich, Zurich, Switzerland; ss Clinical and Experimental Sciences and Human Development in Health, Faculty of Medicine, University of Southampton, Southampton, England; tt Unité de Rhinologie-Olfactologie, Service d’Oto-Rhino-Laryngologie et de Chirurgie cervico-faciale, Hôpitaux Universitaires de Genève, Geneve, Switzerland; uu Division of Otolaryngology-Head & Neck Surgery, Brigham and Women’s Hospital, Harvard Medical School, Boston, MA; vv Royal National Ear, Nose, Throat and Eastman Dental Hospital, UCLH, London, England; ww Skin and Allergy Hospital, Helsinki University Hospital, Helsinki, Finland; xx University of Helsinki, Helsinki, Finland; yy The Allergy Clinic, Johannesburg, South Africa; zz Department of Clinical Science and Education Södersjukhuset, Karolinska Institutet and Sachs’ Children and Youth Hospital, Stockholm, Sweden; aaa Rhinology Unit and Smell Clinic, ENT Department, Hospital Clínic Barcelona, IDIBAPS, Universitat de Barcelona, CIBERES, Barcelona, Catalonia; bbb Pediatric Pulmonology & Allergy Unit, Health Research Institute, La Fe University Hospital, Valencia, Spain; ccc Respiratory Medicine Unit and Oxford Respiratory NIHR BRC, Nuffield Department of Medicine, University of Oxford, Oxford, England; ddd Department of Medicine, Division of Allergy and Immunology, Northwestern University Feinberg School of Medicine, Chicago, IL; eee Observational and Pragmatic Research Institute, Singapore, Singapore; fff Centre of Academic Primary Care, Division of Applied Health Sciences, University of Aberdeen, Aberdeen, Scotland; ggg Department of Allergy, La Paz University Hospital, IdiPAZ, Madrid, Spain; hhh Usher Institute, University of Edinburgh, Edinburgh, Scotland; iii Skåne University Hospital, Department of Otorhinolaryngology, Lund, Sweden; jjj Department of Dermatology, High Altitude Clinic, Medicine Campus Davos-Wolfgang, Davos-Wolfgang, Switzerland; kkk Department of Otorhinolaryngology-Head & Neck Surgery, Vienna, Austria; lll Department of Pediatrics and Adolescent Medicine, Division of Pediatric Pulmonology, Allergology and Endocrinology, Comprehensive Center Pediatrics, Medical University of Vienna, Vienna, Austria; mmm Division of Rhinology, Allergy, and Endoscopic Skull Base Surgery, University of North Carolina at Chapel Hill, Chapel Hill, NC; nnn Patient Advisory Board, The European Forum for Research and Education in Allergy and Airway diseases, Brussels, Belgium; ooo School of Medicine, Griffith University, National Centre for NeuroImmunology and Emerging Disease, Gold Coast, QLD, Australia; ppp Menzies Centre, National Centre for NeuroImmunology and Emerging Disease, Gold Coast, QLD, Australia; qqq Department of Medicine, National Jewish Health, Denver, CO; rrr Novo Holdings, Hellerup, Denmark; sss LungenClinic Grosshansdorf (member of the German Center for Lung Research [DZL]), Airway Research Center North (ARCN), Grosshansdorf, Germany; ttt Christian-Albrechts University, DZL, ARCN, Kiel, Germany; uuu Clinical Department of Otorhinolaryngology, Head and Neck Surgery, University Hospitals Leuven, Leuven, Belgium; vvv World Health Organization, Genèva, Switzerland

**Keywords:** action plan, chronic respiratory diseases, EUFOREA, global health, respiratory care

## Abstract

Chronic respiratory diseases (CRDs) remain 1 of the leading causes of preventable morbidity and disability worldwide, affecting up to one-third of the total Western population in 2025. Recognizing the substantial burden of inflammatory airway diseases such as asthma, COPD, chronic rhinosinusitis, and respiratory allergy, the European Forum for Research and Education in Allergy and Airway Diseases (EUFOREA) organized the symposium “Shaping the Future of Respiratory Care” in April 2025 in Brussels, Belgium, at the occasion of the 10-year jubilee. Featuring keynote speakers from the World Health Organization and EUFOREA, this initiative had the following aims: (1) promoting dialogue on translating innovations into daily clinical practice; (2) encouraging collaboration between the different stakeholders in the respiratory field; and (3) defining strategic priorities to transform respiratory care and arrest the CRD epidemic over the next decade. The symposium highlighted the importance of moving toward predictive, preventive, and patient-centered medicine, while supporting value-based health care systems to improve long-term patient outcomes. This report summarizes the main insights and strategic directions discussed at the meeting.

Chronic inflammatory respiratory diseases (CRDs), including asthma, COPD, chronic rhinitis, and chronic rhinosinusitis (CRS), represent a major and underrecognized global public health challenge, reaching epidemic proportions.^1^^,^^2^ These conditions affect approximately one-third of the world’s population, with increasing prevalence allied to health care expenditure and losses in economic productivity.^3^ In Europe, the annual economic cost of lower airway diseases is estimated at €380 billion, even without accounting for the impact of upper airway diseases.^4^ Despite this, CRDs receive only a small portion (0.1%) of the 2021-2027 EU4Health budget,^5^ indicating a vast gap between the high prevalence and the limited resources allocated.

The European Forum for Research and Education in Allergy and Airway diseases (EUFOREA) symposium entitled “Shaping the Future of Respiratory Care” was held on April 23, 2025, in Brussels, Belgium, to address the urgent need for action. By bringing together diverse perspectives from experts in respiratory medicine, patient advocacy, and public health policy, the goal was to develop practical strategies for improving respiratory health care and fostering innovation in Europe and beyond, building further on the political advocacy symposia organized by EUFOREA in the European Parliament.^3^^,^^6^^,^^7^ The current report details the ambitions and strategic priorities for better care and prevention of CRDs. Ethics committee or institutional review board approval and/or informed consent were not applicable for this work.

## Current Shortcomings of Respiratory Care

Current approaches to respiratory health care reveal major shortcomings that hinder optimal patient care from a medical, societal, and preventive perspective.

A key unmet need remains the early and accurate diagnosis of CRDs. Diagnostic delays, often lasting several years, are common across CRDs, often due to low awareness^8^ and the absence of appropriate diagnostic workup.^9^ In primary care, there are multiple obstacles to overcome. These range from misdiagnosis of asthma^10^^,^^11^ to failure to refer.^12^ However, given that some 25% to 30% of patients attending tertiary level severe asthma clinics do not have asthma (having been already seen by respiratory specialists in secondary care),^13^ this is not a problem isolated to primary care. There is a clear need to identify those with COPD sooner to implement early management, especially smoking cessation. Unfortunately, screening programs are resource intensive, especially in terms of time with a low yield. This is further complicated by the use of different screening techniques, variability of diagnostic criteria applied, availability of appropriate technology, and lack of incentivization.^14^ For asthma and COPD, the limited use of spirometry and other disease prioritization further result in significant diagnostic delays.^15^

Upper airway diseases such as CRS and chronic rhinitis are frequently misdiagnosed or under-recognized,^9^ leading to prolonged patient suffering and increased risk of disease worsening.^16^ However, this is a barrier to care in both primary and secondary care.^17^ A previous EUFOREA initiative showed that most patients with CRS have their first diagnosis with nasal endoscopy after > 5 years of symptom development.^18^ This is despite compelling data showing the utility of proactive diagnosis and treatment, with preventive potential of early diagnosis.^19^^,^^20^

Rhinitis, both allergic and nonallergic, has not been prioritized as a research area by ear, nose, and throat specialists,^21^ making it difficult to know where to refer patients, especially for areas such as immunotherapy or occupational rhinitis, both specialist areas. Patients trust their pharmacists and general practitioners for information and advice on rhinitis.^22^ However, general practitioners self-assess a large need for further knowledge and skills in the management of rhinitis,^23^ and studies in a pharmacy setting have revealed issues such as suboptimal treatment choices, poor adherence, and faulty administration technique.^24^^,^^25^ These observations call for coordinated efforts across all levels of care to enhance patient outcomes.

A further significant gap is the lack of a systematic multidisciplinary approach to care.^26^ The (historical) separation between upper and lower airway disease management has resulted in siloed treatment pathways and missed opportunities for integrated care of “global” airways. In this context, the pocket guides of asthma, CRS, and allergic rhinitis by EUFOREA highlight the importance of multidisciplinary approaches in these (often overlapping) chronic conditions.^27^, ^28^, ^29^, ^30^ Fragmented referral systems and ambiguous referral criteria, alongside poor communication between primary and specialist care, further contribute to delays and suboptimal management.^31^

Many of these poor outcomes are multifactorial in nature. They arise from a lack of sufficient communication between patients and health care providers (eg, due to increasing administrative burden and limited consultation time), lack of point-of-care tools (eg, screening and referral tools), and inadequate resources to sustainably support multidisciplinary care and structured patient follow-up.^31^^,^^32^

**Figure 1:**
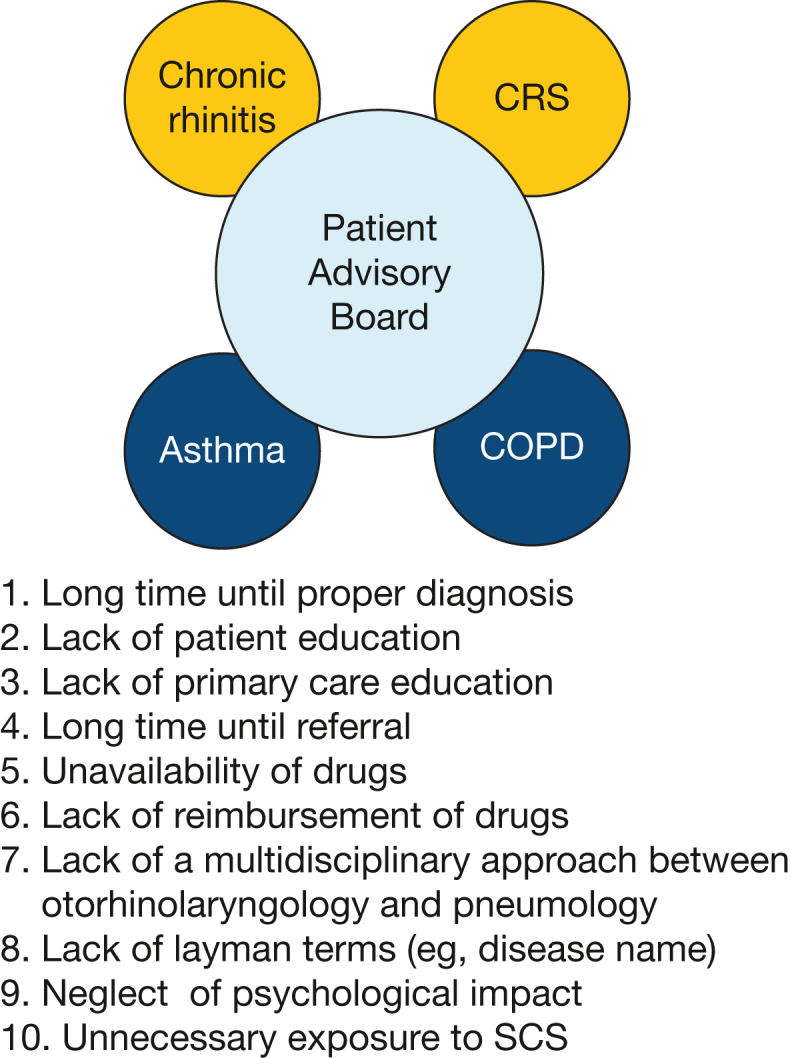
Ten most frequent shortcomings of care expressed by the EUFOREA Patient Advisory Board. CRS = chronic rhinosinusitis; EUFOREA = European Forum for Research and Education in Allergy and Airways diseases; SCS = systemic corticosteroids.

The EUFOREA Patient Advisory Board, with 75 patients living with CRDs, corroborates these shortcomings of care (Fig 1). Their (anecdotal) experiences point to unmet needs such as limited patient education tools, limited collaboration between different medical specialties, unnecessary exposure to systemic corticosteroids, and restricted access to advanced therapies such as biologic agents for severe asthma and CRS with nasal polyps (CRSwNP). These deficiencies result in inefficient care and overall suboptimal health outcomes.^33^^,^^34^

## Predictive and Preventive Respiratory Medicine

**Figure 2:**
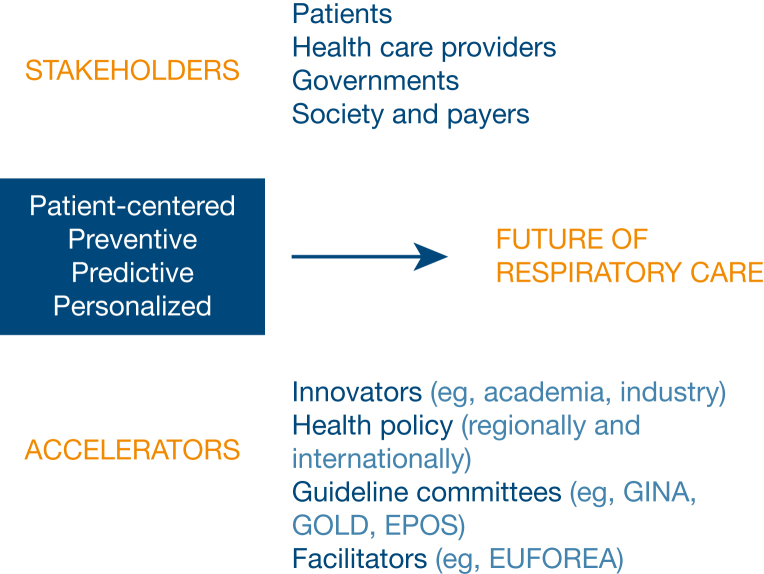
Shaping the future of respiratory care by different stakeholders and accelerators. EPOS = European Position Paper on Rhinosinusitis and Nasal Polyps; EUFOREA = European Forum for Research and Education in Allergy and Airways diseases; GINA = Global Initiative for Asthma; GOLD = Global Initiative for Chronic Obstructive Lung Disease.

A key theme of the symposium was the necessity of moving toward patient-centered, preventive, predictive, and personalized respiratory care (Fig 2, Table 1). Research shows that many CRDs share biological pathways, often involving type 2 inflammation (eg, asthma, CRSwNP, allergic rhinitis, a subgroup of COPD), with characteristic markers such as increased expression of the type 2 cytokines IL-4, IL-5, and IL-13, as well as alarmins, eosinophilia, and elevated IgE.^35^^,^^36^ Biomarkers such as blood/sputum eosinophil counts, fractional exhaled nitric oxide, serum periostin, serum total IgE, and nasal polyp tissue eosinophilia are proving to be helpful in secondary/tertiary care clinics to guide treatment in a personalized and predictive way, leading to better outcomes of care.^37^, ^38^, ^39^

**Table 1:** Strategic Recommendations for Transforming Respiratory Care

Domain	Current Gaps	Recommended Actions	Expected Outcomes
Health care systems	Diagnostic delays and fragmented care pathways	Implement evidence-based screening programs	Earlier diagnosis of at-risk patients and first-time-right treatment initiation
	Limited and unequal access to essential and innovative therapies (eg, biologic agents)	Establish integrated multidisciplinary care pathways across pulmonology, otorhinolaryngology, allergology, and primary care	Reduced hospitalizations and more efficient use of health care resources
	Poor referral coordination and transitions between primary, secondary, and tertiary care	Implement reimbursement policies ensuring equitable timely access to evidence-based therapies	Improved patient outcomes and quality of life, and greater health equity
		Deploy digital tools for systematic monitoring and follow-up	
Clinical guidelines	Reactive, symptom-based treatment paradigms	Prioritize remission end points and de-escalation protocols	Reduced systemic corticosteroid and SABA burden
	Lack of multimorbidity and global airway concepts	Define phenotypic and endotypic classification with validated biomarker thresholds across CRDs	Enhanced secondary and tertiary prevention
	Suboptimal implementation of key guideline messages (eg, OCS and SABA-sparing strategies)	Shift toward biology-informed treatment algorithms	Accelerated time-to-remission through biomarker-guided management
Research priorities	Underfunding of CRD research	Increase funding for prevention, early intervention, and translational research	Identification of predictive modifiable risk factors
	Limited understanding of life-long disease trajectories and precursor stages	Advance predictive biomarkers discovery for disease activity and therapeutic response (eg, omics-based profiling)	Evidence-based prevention strategies
	Limited real-world effectiveness data and head-to-head trials	Generate RWE on remission, disease modification, and long-term outcomes	Stronger support for personalized and predictive care models
	Uncertainty regarding optimal care pathways	Generate health economic evidence to support optimized research allocation	Health economic justification for earlier, more targeted interventions

CRD = chronic respiratory diseases; OCS = oral corticosteroid; SABA = short-acting beta-agonist; RWE = real-world evidence.

However, state-of-the-art guidelines still consider a stepwise escalation of treatment based on phenotypic worsening of the disease and clinical symptoms, with little attention given to biology-informed and biomarker-guided treatment decisions.^40^ This is a reflection of the current practices in most clinical centers worldwide, while emerging evidence supports new possibilities to optimize the patient journey, with more personalization of care.^41^ Through a better understanding of pathophysiological mechanisms, patient’s identified needs and concerns, and associated disease biomarkers, disease development might be halted early or attenuated over time.^3^^,^^6^^,^^40^^,^^42^^,^^43^

A paradigm shift will be needed in the decade to come with a focus on the primary prevention of CRDs in healthy individuals, while targeting remission in patients through disease modification.^44^ The ambition of remission and even cure requires further acceleration of basic and translational research with appropriate and personalized predictive implementation plans.^35^^,^^44^, ^45^, ^46^ Indeed, personalized treatment plans based on biology have been largely unexplored and still show high potential for improving outcomes and reducing the use of health care resources.^41^ Given the high costs of novel therapeutic approaches with disease-modifying and preventive potential, the need for *precision medicine* has become urgent and in high demand.

Advances in multi-omics profiling could help predict/select patients who are most likely to develop severe and/or irreversible CRDs and who might benefit from (early) targeted therapies.^47^ Identifying genetic and epigenetic markers of CRDs has also proved useful for streamlining drug development and repurposing, and might offer new possibilities for personalized regimens in the future.^48^ Putting these into practice requires prioritization of said research through sustainable funding, educational efforts to train health care providers with the latest innovations, and adaptive reimbursement policies to reflect and expedite these changes in clinical practice.

During this new era of respiratory care, the application of digital health tools (eg, digital twins and artificial intelligence [AI]-driven support systems) is expected to be a key part of patient management.^49^ Indeed, telemonitoring and mobile applications can support adherence, self-management, and earlier diagnosis.^50^ There are already good frameworks in place,^51^ but there are many challenges ahead in terms of motivation and methodologies,^52^ not to mention the challenges of adequate governance. Patients will need to be involved in the design of any application alongside practicing clinicians to ensure that any app is fit for purpose in line with design principles.^53^ A full roll-out in the future could also allow for more spatiotemporal (exposure) data collection, potentially enabling predictive analytics.^54^ Thus, guideline development committees need to be prepared to capture and translate the increasing complexity and further individualization of care into clinical care pathways that allow for earlier treatment and proactive disease management.

AI holds significant promise to transform respiratory care by improving diagnostic accuracy, supporting personalized treatment decisions, and identifying patients at risk of deterioration.^55^ AI-driven models can integrate imaging, biomarkers, and patient-reported outcomes to guide earlier and more precise interventions. To be clinically useful, however, such tools must be explainable, validated across diverse populations, and embedded into existing workflows without adding administrative burden.^32^ Used in this way, AI can enhance clinician decision-making and support more meaningful patient-provider interactions at point-of-care.

## Building Collaborative and Patient-Centric Care

A key limiting factor is that effective respiratory care requires collaboration across different medical disciplines. The symposium strongly emphasized the need for more integrated, multidisciplinary care models across sub-specialties, including primary care, pulmonology, otorhinolaryngology, allergology, pediatrics, psychology, and rehabilitation services. Furthermore, smooth transitions and bidirectional communication between different levels of care (primary, secondary, and tertiary) are essential for optimizing outcomes and efficient use of finite resources.^31^ More initiatives aiming to facilitate this approach, such as the EUFOREA multidisciplinary pocket guides^30^ and multidisciplinary training courses^56^ uniting different specialists, are highly welcomed and adopted by centers worldwide. Other educational platforms, including the European Respiratory Society learning modules and Harmonised Education in Respiratory Medicine for European Specialists (HERMES) examinations, the American Academy of Allergy, Asthma and Immunology certifications, and American College of Chest Physicians’ recent exploration of a certification program for advanced practice providers in pulmonary medicine, show the growing ecosystem of respiratory education needed for an effective workforce.

Furthermore, actively making the patient the center of their journey is equally important. Patients who are well informed about their condition and treatment tend to follow their therapeutic plans more closely and have better outcomes.^57^, ^58^, ^59^, ^60^ A key approach is to empower patients by education and supported self-management^61^ to strengthen them in advocating for their own health. Indeed, better patient education can lead to better treatment adherence, fewer exacerbations, and improved quality of life.^62^^,^^63^ EUFOREA’s Patient Advisory Board ensures that patient perspectives are being represented through advocacy efforts and are involved bottom-up in educational materials and research projects.^33^^,^^34^^,^^45^ Furthermore, particularly relating to translational health issues, patients need to be actively involved in the design of prospective trials.^64^

## Health Policy for Systemic Change

To help transform respiratory health care, including the aforementioned recommendations, strong political support and proactive public health policies are essential. Giving CRDs greater recognition within the public health agenda is necessary to secure adequate funding, effective prevention strategies, and rapid access to advanced treatments when clinically indicated. Access to both essential and advanced therapies continues to vary greatly across countries, further compromising health equity. The upcoming Fourth High-Level Meeting of the UN General Assembly on preventing and controlling noncommunicable diseases (NCDs)^65^ is an important opportunity to encourage global action and include CRD management within broader NCD frameworks, building on initiatives such as the EU NCD initiative.^66^ The need for prevention has been a key strategic focus area of EUFOREA since its foundation in 2015.^3^^,^^7^

Historical measures, such as strengthening tobacco control, have shown a substantial positive impact on the burden of CRDs and public health more broadly.^67^ Further real-world evidence on preventive and therapeutic interventions could help allocate resources more effectively. The opportunity cost of inaction is not only medically and morally unacceptable but also undermines the prosperity of societies in all regions.^68^ Indeed, integrated public health strategies that combine preventive measures (eg, mitigating indoor and outdoor atmospheric pollution) with an optimization of current and future clinical care pathways, while safeguarding clinical autonomy for more personalized care, could reduce respiratory illness and related costs.^69^

Furthermore, global collaboration and partnerships are vital. Disparities in care are still abundant in 2025 worldwide due to varying resources, infrastructure, and priorities.^70^ EUFOREA’s strategic priorities align with and complement ongoing initiatives by the World Health Organization and guideline committees (Global Initiative for Asthma, the Global Initiative for Chronic Obstructive Lung Disease, and the European Position Paper on Rhinosinusitis and Nasal Polyps, Allergic Rhinitis and Its Impact on Asthma). More specifically, EUFOREA’s contribution is in bridging the latest scientific insights into clinical practice by providing clinically relevant guidance through multidisciplinary education and practical decision-support tools. Ongoing challenges in clinical management (eg, inappropriate use of [repeated] oral steroids and antibiotics, short-acting beta-agonists without inhaled corticosteroids in asthma, repeated polypectomies in CRSwNP) remain common and warrant a coordinated, global, and cross-disciplinary action. Having a highly ambitious framework (eg, targeting prevention and remission) will also help in identifying the lessons learned from leading and lagging health care systems.^6^

Moving toward value-based health care, which focuses on outcomes relative to costs for each stakeholder, might also increase the likelihood of long-term investment in CRD care.^71^ Strong real-world evidence and economic analyses are welcomed to corroborate and prioritize early diagnosis, optimized treatment, and prevention. Although this symposium focused on asthma, COPD, CRS, and allergic rhinitis, many of the systemic-level recommendations are broadly applicable across respiratory medicine. Specifically, the health care system reforms outlined in Table 1 (integrated multidisciplinary care pathways, evidence-based screening programs, and digital monitoring tools) represent infrastructure improvements that will also indirectly benefit patients with restrictive lung diseases, sleep-related breathing disorders, and other chronic respiratory conditions.

Although provider qualifications were not discussed during the symposium, they remain essential for implementing modern respiratory care. As diagnostics and treatment pathways become more complex, harmonized and Continuing Medical Education-accredited training is critical to ensure a competent workforce across all levels of care. International alliances such as the Biomedical Alliance in Europe play a critical policy role in promoting consistent, high-quality educational standards.

## EUFOREA’s Action Plan

**Figure 3:**
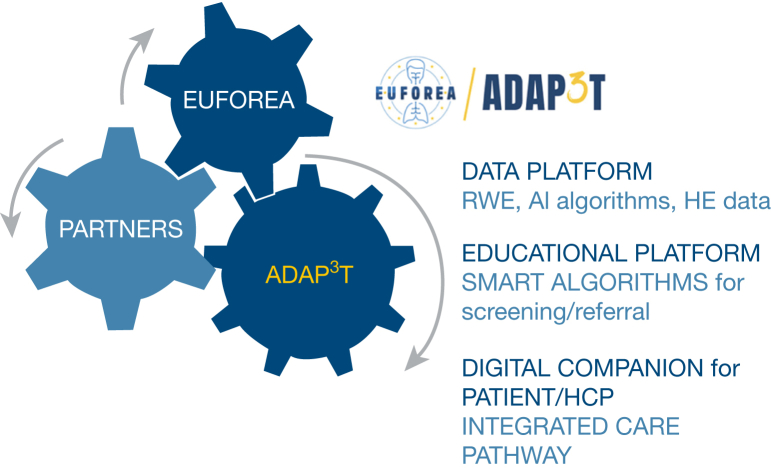
Positioning of ADAP^3^T to facilitate multistakeholder collaboration and develop practical tools to address unmet needs by the different stakeholders. ADAP^3^T = Airways Disease Action Plan for Personalised and Preventive Treatment; AI = artificial intelligence, HCP = health care professional; HE = health economics; RWE = real-world evidence.

With remission now recognized^44^ as a most ambitious treatment goal in CRSwNP and asthma, all stakeholders and their representatives must join forces to achieve remission in a substantial proportion of patients experiencing inflammatory CRDs.^40^ EUFOREA’s flagship project, the Airways Disease Action Plan for Personalised and Preventive Treatment (ADAP^3^T), is a multistakeholder and collaborative plan to help arrest the growing epidemic of CRDs through a digital companion for patients and clinicians, aiding in overcoming the current obstacles for the implementation of preventive strategies and optimal use of resources (Fig 3).

ADAP^3^T will encompass a digital companion through which patients are actively supported in reaching better outcomes of care through AI-driven support, based on big health data insights and guideline-based algorithms. It capitalizes on opportunities such as self-education and remote patient (self-) monitoring to facilitate optimal clinical pathways.^18^^,^^49^ Current technologies indeed allow for collecting real-time data and personalized notifications. However, specific tools addressing the most common CRDs are largely lacking or inadequate. By taking into account the lessons learned from the last decade, there is a huge potential for these tools to reduce diagnostic delays, improving personalized, predictive, and preventive therapy, and making referrals more efficient. Particularly, in the context of primary care, in which the large majority of patients are being managed, tools that inform and enhance effective management are worthwhile and badly needed by clinicians and patients alike.^72^

Digital health tools such as the planned ADAP^3^T framework should support the strategic pillars of predictive, preventive, personalized, and patient-centric care to enable practical implementation. In the ADAP^3^T concept, the predictive component would help flag patients at risk of uncontrolled disease; the preventive component would support earlier intervention through risk stratification; the personalized component would tailor recommendations to individual symptom patterns and biomarker profiles; and the patient-centric component would facilitate shared decision-making and self-management through educational tools of EUFOREA.

Tools that optimize and streamline the workflows of health care providers might also prove to be beneficial to improve evidence-based care,^41^ while integrating continuous medical education. Keeping up-to-date with evolving precursor stages of disease (eg, pre-COPD, pre-asthma),^43^^,^^73^ treatment goals (eg, control and remission),^40^^,^^44^ and innovative therapies (eg, biologic agents)^37^^,^^74^ is key to strengthening patient care. EUFOREA will support these efforts through the development of digital education platforms with interactive modules and decision support tools to reduce disparities in health equity and improve outcomes.^70^

## Conclusions

This EUFOREA jubilee symposium highlighted the urgent need to transform the management of CRDs with a focus on prevention, early diagnosis, and patient-centered care. Key strategies involve adopting predictive and preventive medicine, implementing integrated care, supporting policies and global collaboration, and embracing digital tools. The next decade requires efficient translation of latest innovations into tangible benefits for patients, working toward a future of proactive respiratory management and improved quality of life worldwide to meet the World Health Organization’s NCD Prevention and Control targets of 2030 and beyond.^75^

## Funding/Support

The “Shaping the Future of Respiratory Care” Symposium was organized without the support of corporate partners of EUFOREA or any other source of funding.

## Financial/Nonfinancial Disclosures

The authors have reported to *CHEST* the following: X. B. reports advisory boards for GSK and Sanofi-Regeneron. G. K. S. reports honoraria from ALK, Bayer, GSK, Haleon, Noucor, Sanofi-Regeneron, and Viatris; advisory boards for ALK, Bayer, GSK, Haleon, Noucor, Sanofi-Regeneron, and Viatris; and leadership roles as Chair of BSACI rhinitis guidelines, Scientific Chief Editor of Frontiers in Allergy Rhinology Section, and Vice President of EUFOREA. S. L. reports honoraria from Allergopharma, DBV Technologies, Sanofi-Aventis, Leti, and Nutricia; and advisory boards for Leo-Pharma, GSK, and Sanofi-Aventis. W. J. F. reports grants to her institution from Novartis, EU, GSK, and Sanofi-Aventis; and consulting fees from Dianosic, GSK, Novartis, Sanofi-Aventis/Regeneron, and AstraZeneca. P. J. B. reports grants from AstraZeneca; and honoraria and advisory boards from AstraZeneca, EpiEndo, InStatin, PulmBioMed, and Boehringer Ingelheim. M. B. S. reports honoraria from Sanofi, GSK, Bionorica, Salvat, and Zenith; travel support from Salvat and Zenith; and grants from Springer. E. B. reports honoraria from Novartis and Sanofi; travel support from EUFOREA; advisory board for ligelizumab; and leadership roles in WAO Atopic Dermatitis Committee and EUFOREA AIT Committee. M. C. B. reports grants from GSK, CK-CARE, AstraZeneca, LEO Foundation, LEO Pharma, and the Swiss National Science Foundation; consulting fees from Sanofi, LEO Pharma, Almirall, AbbVie, and GSK; and honoraria from Sanofi, AbbVie, Almirall, and GSK. G. G. B. reports honoraria from AstraZeneca, Boehringer Ingelheim, Chiesi, GSK, Sanofi, and Regeneron. D. M. C. reports nonfinancial interests as Academic Manager at EUFOREA and Associate Editor at Frontiers in Allergy. M. E. C. reports grants from Sanofi-Regeneron; consulting fees from GSK and Sanofi-Regeneron; honoraria from GSK and Sanofi-Regeneron; travel support from Sanofi; and a leadership role at EUFOREA. E. D. C. reports honoraria from GSK, Sanofi, AstraZeneca, Regeneron, Firma, and Novartis. R. D. reports consulting fees and stock ownership in Synairgen. A. T. F. reports grants from BBC and ITV; consulting fees from Viatris and ALK-Abello; honoraria from Aimmune; stock in AllergyRhino; and leadership as Chair of the National Allergy Strategy Group UK. M. G. reports travel support from Menarini. P. G. reports grants from Sanofi and GSK; consulting fees from Lilly, Insmed, and AstraZeneca; honoraria from GSK and Sanofi; and travel support from GSK. C. G. reports honoraria from Danone, Aspen, Viatris, and Inova. J. H. reports consulting fees from Sanofi, Regeneron, GSK, and AstraZeneca. L. G. H. reports grants from MedImmune, Novartis UK, Roche/Genentech, GSK, Amgen, AstraZeneca, Aerocrine, and Vitalograph; honoraria from Amgen, AstraZeneca, Boehringer Ingelheim, Chiesi, Circassia, Hoffmann-La Roche, GSK, Novartis, Theravance, Evelo Biosciences, Sanofi, and Teva; travel support from AstraZeneca, Boehringer Ingelheim, Chiesi, GSK, and Napp Pharmaceuticals; and advisory boards for Amgen, AstraZeneca, Boehringer Ingelheim, Chiesi, Circassia, Hoffmann-La Roche, GSK, Novartis, Theravance, Evelo Biosciences, Sanofi, Teva, and Janssen. E. H. reports grants from Chiesi; consulting fees from Chiesi, Almirall, Bosch Healthcare, GSK, Celltrion Healthcare, and Apogee Therapeutics; honoraria from AstraZeneca, Sanofi, GSK, Chiesi, Firma, Gentili, Lofarma, and Orion Pharma; and advisory boards for AstraZeneca, Sanofi, Regeneron, GSK, Celltrion Healthcare, Allergy Therapeutics, and Blueprint Medicines. C. H. reports honoraria from Sanofi, AstraZeneca, and GSK; advisory boards for Sanofi, AstraZeneca, Lilly, GSK, and Medtronic. D. J. reports grants from the National Institute of Allergy and Infectious Diseases, the National Heart, Lung, and Blood Institute, the National Institutes of Health, GSK, and Regeneron; consulting fees from Avillion, AstraZeneca, GSK, Genentech, Regeneron, Sanofi, and OM Pharma; and Data and Safety Monitoring Board for Pfizer. M. J. reports honoraria from Stallergenes Greer, Takeda, ALK, GSK, Novartis, AstraZeneca, Chiesi, Viatris, and Sanofi; and advisory boards for Takeda, AstraZeneca, GSK, Berlin-Chemie Menarini, Viatris, and KalVista. S. L. reports consulting fees from AstraZeneca, Genentech, GSK, Lyra Therapeutics, OptiNose, and Sanofi-Regeneron. V. L. reports consulting fees from GSK and AstraZeneca; and honoraria from Abbott, Novartis, and Sanofi. E. M. reports honoraria from Airsonett, ALK, AstraZeneca, Chiesi, Novartis, and Sanofi; and advisory boards for Airsonett, ALK, AstraZeneca, Chiesi, Novartis, and Sanofi. J. M. reports grants from Viatris/MEDA Pharma and the Noucor/Uriach Group; honoraria from Menarini, GSK, MSD, Mitsubishi-Tanabe, Noucor/Uriach Group, Viatris/MEDA Pharma, AstraZeneca, Sanofi-Genzyme, and Regeneron Pharmaceuticals; expert testimony for Uriach Group and Sanofi; and advisory boards for Sanofi-Genzyme, AstraZeneca, Regeneron Pharmaceuticals, Almirall, Viatris/MEDA Pharma, Noucor/Uriach Group, GSK, and Lilly. I. P. reports grants from Chiesi; consulting fees from Almirall, AstraZeneca, Boehringer Ingelheim, Chiesi, Circassia, Dey Pharma, Genentech, GSK, Knopp Biosciences, Merck, MSD, Napp Pharmaceuticals, Novartis, Regeneron Pharmaceuticals, RespiVert, Sanofi, Schering-Plough, and Teva Pharmaceuticals; honoraria from Aerocrine AB, Almirall, AstraZeneca, Boehringer Ingelheim, Chiesi, GSK, Novartis, Regeneron Pharmaceuticals, Sanofi, and Teva Pharmaceuticals; and travel support from AstraZeneca, Boehringer Ingelheim, Chiesi, GSK, Napp Pharmaceuticals, Regeneron Pharmaceuticals, Sanofi, and Teva Pharmaceuticals. A. P. reports grants from AstraZeneca, Sanofi-Regeneron, Insmed, and Aretia; and consulting fees from AstraZeneca, Sanofi-Regeneron, Chiesi, GSK, and Eli Lilly. D. P. reports grants from AstraZeneca, Chiesi, Viatris, Novartis, Regeneron Pharmaceuticals, Sanofi-Genzyme, and UK NHS; consulting fees from AstraZeneca, Boehringer Ingelheim, Chiesi, GSK, Novartis, Teva Pharmaceuticals, and Viatris; honoraria from AstraZeneca, Boehringer Ingelheim, Chiesi, Cipla, Inside Practice, GSK, Novartis, Medscape, Regeneron Pharmaceuticals, Sanofi-Genzyme, Teva Pharmaceuticals, and Viatris; expert testimony for GSK; travel support from AstraZeneca, Boehringer Ingelheim, Novartis, Medscape, and Teva Pharmaceuticals; advisory boards for AstraZeneca, Amgen, Boehringer Ingelheim, Chiesi, Novartis, Regeneron Pharmaceuticals, Sanofi-Genzyme, Teva Pharmaceuticals, and Viatris; stock ownership in Optimum Patient Care Ltd and Observational and Pragmatic Research Institute Pte Ltd. S. Q. reports consulting fees from GSK; honoraria from AstraZeneca, GSK, Chiesi, Allergy Therapeutics, Regeneron, Sanofi, and Gebro; and travel support from AstraZeneca and GSK. D. R. reports honoraria from ALK-Abello, Menarini, and Thermo Fisher; travel support from Chiesi and IPCRG; and a leadership role at IPCRG. S. Scheire reports grants from EUFOREA. S. Schneider reports grants from AstraZeneca, Sanofi, and GSK; honoraria from AstraZeneca, Sanofi, and GSK; expert testimony for GSK; travel support from AstraZeneca, Sanofi, and GSK; and advisory boards for AstraZeneca, Sanofi, and GSK. B. A. S. reports consulting fees from Lyra, Stryker, Neurent, Spirair, and MCP. M. E. W. reports consulting/advisory honoraria from Allakos, Areteia Therapeutics, Arrowhead Pharmaceutical, Avalo Therapeutics, Belenos Bio, Celldex, Connect Biopharma, Eli Lilly, Enveda Therapeutics, Equillium, General Medicines, Jasper Therapeutics, Kinaset, Kymera, Merck, MyBiometry, Pharming, Phylaxis, Pulmatrix, Rapt Therapeutics, Recludix Pharma, Roche/Genentech, Sentien, Sound Biologics, Tetherex Pharmaceuticals, Uniquity Bio, Verona Pharma, Zura Bio, AstraZeneca, Amgen, Regeneron, GSK, and Sanofi-Genzyme; research support from AstraZeneca, Amgen, Regeneron, GSK, Sanofi-Genzyme, and Upstream Bio; and stock options in Cellergy Pharma Inc, and Upstream Bio. K. F. R. reports consulting fees from AstraZeneca, Boehringer Ingelheim, Chiesi, and Sanofi-Regeneron; honoraria from AstraZeneca, Boehringer Ingelheim, Chiesi Pharmaceuticals, CSL Behring, Sanofi-Regeneron, GSK, Berlin Chemie, and Menarini; advisory boards for AstraZeneca, Boehringer Ingelheim, Sanofi-Regeneron, and CSL Behring; leadership roles at the German Center for Lung Research (DZL), the German Chest Society (DGP), and the American Thoracic Society. P. W. H. reports grants from Sanofi-Regeneron, Novartis, GSK, Medtronic, and Viatris; consulting fees from Sanofi-Regeneron, Novartis, GSK, Medtronic, and Viatris; and honoraria from Sanofi-Regeneron, Novartis, GSK, Medtronic, and Viatris. None declared (V. B., L. B., M. S. B., L. O. C., B. D. G., P. G. G., H. J. H., O. J., P. J., E. K., B. N. L., M. Mäkelä, M. McDonald, A. N. G., P. S.-J., P. S.-G., C. M. E. S., P. K. S., Z. S., M. T. A. T., P. H., J. L. C.).
